# Immobilization of Heavy Metals in Boroaluminosilicate Geopolymers

**DOI:** 10.3390/ma14010214

**Published:** 2021-01-04

**Authors:** Piotr Rożek, Paulina Florek, Magdalena Król, Włodzimierz Mozgawa

**Affiliations:** Faculty of Materials Science and Ceramics, AGH University of Science and Technology, 30 Mickiewicza Av., 30-059 Krakow, Poland; paulina@agh.edu.pl (P.F.); mkrol@agh.edu.pl (M.K.); mozgawa@agh.edu.pl (W.M.)

**Keywords:** alkali-activation, fly ash, boron, lead, nickel, leaching

## Abstract

Boroaluminosilicate geopolymers were used for the immobilization of heavy metals. Then, their mechanical properties, phase composition, structure, and microstructure were investigated. The addition of borax and boric acid did not induce the formation of any crystalline phases. Boron was incorporated into the geopolymeric network and caused the formation of N–B–A–S–H (hydrated sodium boroaluminosilicate) gel. In the range of a B/Al molar ratio of 0.015–0.075, the compressive strength slightly increased (from 16.1 to 18.7 MPa), while at a ratio of 0.150, the compressive strength decreased (to 12 MPa). Heavy metals (lead and nickel) were added as nitrate salts. The loss of the strength of the geopolymers induced by heavy metals was limited by the presence of boron. However, it caused an increase in heavy metal leaching. Despite this, heavy metals were almost entirely immobilized (with immobilization rates of >99.8% in the case of lead and >99.99% in the case of nickel). The lower immobilization rate of lead was due to the formation of macroscopic crystalline inclusions of PbO·xH_2_O, which was vulnerable to leaching.

## 1. Introduction

Rapidly growing industries have given humanity ever higher standards of living, but improper management can have serious consequences for the environment. One of the most important impacts is environmental pollution, such as greenhouse gas emissions to the atmosphere, the formation of heaps from the deposit of solid wastes, and the contamination of water and soil with heavy metals. In order to protect the environment, some restrictions have been imposed and some goals have been established in order to create a “recycling society” that seeks to eliminate waste generation and use it as secondary raw material. Ordinary Portland cement (OPC) is one of the most used materials in the world, but it has a large carbon footprint, so research into obtaining its ecological alternative is obviously needed.

Geopolymers, like alkali-activated aluminosilicates, are defined as binder materials built from a tri-dimensional network structure of –Si–O–Si(Al)– bonds and synthesized by treating the reactive solid source of SiO_2_ and Al_2_O_3_ with an alkaline solution [1]. Due to their similarity in chemical structure and physical properties to hardened ordinary Portland cement, geopolymers are considered a green alternative of the most commonly used building material in the world [2]. The relatively low alkali activation temperature of aluminosilicates (<90 °C), along with the lack of need for calcine substrates and fuse clinkers, results in reduced energy consumption and a lower amount of CO_2_ released into the atmosphere in the case of geopolymers in comparison with OPC. The final solid products of alkali-activated aluminosilicates are highly chemical and fire-resistant, and they exhibit relatively high mechanical strengths [3]. Therefore, the physical properties of geopolymers show their potential to be environmentally-friendly Portland cement alternatives that can be used as construction and building materials. There are various known reactive solid sources of SiO_2_ and Al_2_O_3_, starting from synthetic pure chemical reagents to natural minerals and industrial by-products. According to the literature, geopolymers have been successfully obtained from coal fly ash [4], bottom ash [5], red mud [6,7], slag [8], and biomass fly ash [9]. This fact suggests an excellent opportunity to reuse materials that are currently waste.

The composition of ashes, generated both in power plants and waste incinerators, depends on the source of solid fuels used in the combustion process, but it usually contains heavy metals. Without treatment, hazardous ions can be washed out and pose a threat to the environment. Heavy metals can get into the soil and water, enter plants, and then pass through the food chain to animal and human bodies. Immobilization is the chemical process of material transformation in such a way that soluble compounds are not eluted. One of the most effective materials for deactivating compounds is glass due to its high chemical resistance [10]. However, such a process is rarely used due to the fact that it is energy-consuming. In the literature, one can find more cost-effective methods with the use of cement pastes [11], slag-alkali binders [12], and red ceramics [13].

Another important aspect of alkali-activated aluminosilicates is their ability to immobilize hazardous ions, which has been widely investigated in the past few years, e.g., the immobilization mechanism of heavy metal cations (Cd^2+^, Pb^2+^, and Zn^2+^) and anions (AsO_4_^3−^ and Cr_2_O_7_^2−^) in the composite geopolymers based on granulated blast furnace slag and drinking water treatment residue [14] or the immobilization of hexavalent chromium in fly ash-based geopolymers [15]. Additionally, the results described in [16] showed a matrix based on alkali-activated aluminosilicates that could be an alternative to cementitious binders. The proposed mechanisms of the heavy metal immobilization in geopolymers involve the ion-exchange of charge-balancing cations (Na^+^) with heavy metal cations; covalent bonding of heavy metals to the aluminosilicate network of a geopolymer; the precipitation of hydroxides, carbonates, silicates of heavy metals; and the physical encapsulation of heavy metals in a low-permeable geopolymeric matrix [17]. Heavy metals may be trapped in closed micropores created in a geopolymeric matrix [18]. When the heavy metal leaching from geopolymers is below the standard limits, the geopolymers can be used in some construction applications [19].

In order to make geopolymers more cost-effective and eco-friendly, some attempts have been made to introduce other elements, such as boron, which could totally or partially substitute alumina or silica. Boron is used in different industries, such as, energy, agriculture, medicine, and glass, detergent, and ceramics manufacturing [20]. It is present in nature in many kinds of minerals, and its main salts (borates) are not considered toxic substances [21]. There have been some studies regarding geopolymers with the addition of various boron compounds, such as borax, anhydrous borax, boric acid, amorphous and crystalline lithium tetraborate, and colemanite [22,23,24,25,26,27,28,29,30]. Boron may also be introduced to a geopolymer matrix when the raw aluminosilicate material contains this element. Celik et al. [20] made geopolymers based on metakaolin and colemanite waste, while Taveri et al. investigated the utilization of recycled borosilicate glass for geopolymer production [31]. The impact of using borax as a component of the alkalizing solution was undertaken in the mentioned literature, and the chemical was considered to be an environmentally-friendly additive that could influence mechanical properties. In Portland cement systems, boron acts as a hydration retarder, and it has a strong impact on hydration kinetics—it slows down the dissolution of aluminate phases [32]. Boron negatively affects the hydration and mechanical strength of Portland cement, but it has no influence on the alkaline activation of fly ashes [21]. However, in geopolymers activated with NaOH and sodium silicate, a decrease in the content of sodium silicate in the activator solution was found to lead to a steep decrease in compressive strength, while the presence of borax allowed for the obtainment of a much slighter decrease in compressive strength [24].

To the best of our knowledge, there have been descriptions of boroaluminosilicate geopolymers utilized as matrixes for the disposal of ashes containing heavy metal ions. Taking into account the nature of boron, its ability to complex various elements, and the commonly known immobilizing properties of borosilicate glasses [33], the immobilizing properties of boroaluminosilicate geopolymers were investigated in the present work. The aim of this research was to utilize alkali-activated fly ash as a matrix for the immobilization of heavy metals and to assess the role of boron in immobilization efficiency. The results of structural studies of boroaluminosilicate geopolymers, obtained via an alkali-activation method of fly ash with two different sources of boron, are also presented. The results show the possibility of the utilization of industrial by-products bearing high amounts of heavy metals and boron compounds for the production of geopolymers that could be used, e.g., in roads basements construction.

## 2. Materials and Methods

Coal fly ash (FA) obtained from a Polish power plant was used as an aluminosilicate source for alkali-activation, which was conducted with a sodium hydroxide solution. Additionally, borax and boric acid were used to introduce boron to the structure of the geopolymers. Lead nitrate, Pb(NO_3_)_2_, and nickel nitrate, Ni(NO_3_)_2_·6H_2_O, were utilized to determine the immobilization properties of the geopolymers.

Geopolymers were obtained by mixing fly ash with an alkali activator (NaOH) solution (AA). Two series with boron were prepared—one with borax (BX), Na_2_[B_4_O_5_(OH)_4_]·8H_2_O and one with boric acid (BA), H_3_BO_3_—as sources of boron. The amount of boric compound represented four molar ratios of boron to aluminum: B/Al = 0.015, 0.030, 0.075, and 0.150 (Table 1). The amount of aluminum in fly ash was determined with the X-ray fluorescence (XRF) method.

The samples with heavy metals were prepared by adding a metal salt to the geopolymeric slurry. The amount of the metal salt represented the amount of metal cations in relation to fly ash, and it was 2 and 4 wt.%. After mixing, the pastes were cast in silicone molds, thus obtaining cubic samples (20 × 20 × 20 mm^3^) that were cured at 80 °C for one day. The samples were tested after another 27 days.

XRF was used for the quantitative analysis of fly ash and geopolymers (with an Axios PANalytical Max 4 kW spectrometer (PANalytical, Malvern, UK) with a wavelength dispersive and Rh source). The standardless analysis package Omnian (PANalytical) was used for the semiquantitative analysis of the spectra. The results are presented on a percentage scale (normalized to 100%). XRD (X-ray diffraction) was used to analyze the phase composition of the samples; a diffractometer (Empyrean, PANalytical) was with CuKα radiation and a graphite monochromator, and the measurements were carried out in the 2theta angle range of 5–60° for 3 h with a step of 0.007. Phases were identified with the use of an X’Pert HighScore Plus (PANalytical) application and the International Centre for Diffraction Data. SEM (scanning electron microscope) was used to observe the microstructure of the samples (with an FEI Nova NanoSEM 200 microscope; samples were sprayed with graphite), to observe the microstructure, and prepare EDS (energy-dispersive X-ray spectroscopy) maps (with ThermoFisher Scientific Phenom XL (ThermoFisher Scientific, Waltham, MA, USA); samples were sprayed with gold). FT-IR (Fourier transform infrared spectroscopy) was used to study the structure of the samples (with a Bruker VERTEX 70v vacuum spectrometer (Bruker, Billerica, MA, USA); samples were prepared as KBr pellets and spectra were measured in the mid infrared range (4000–400 cm^−1^) with 128 scans and a resolution of 4 cm^−1^). The bulk densities were calculated by dividing the mass of the sample by its volume. The compressive strength of each sample was measured with a ZwickRoell (Ulm, Germany) machine working on the principle of a hydraulic press.

The leaching test was prepared according to the procedure described in the EN 12457-2:2002 (Characterization of Waste—Leaching) standard. The geopolymer samples were crushed, and the size of the particles for the leaching test was below 4 mm. They were poured with distilled water and shaken for 24 h. The ratio of the leaching solution (L) to the sample (S) was: L/S = 10 L/kg. The concentrations of Pb^2+^ and Ni^2+^ cations in the leachates were analyzed by atomic absorption spectrometry (AAS). The analyses were conducted on a Philips PU 9100× spectrometer (Philips, Amsterdam, The Netherlands) with a calibration curve.

## 3. Results

### 3.1. Characterization of Fly Ash

**Figure 1:**
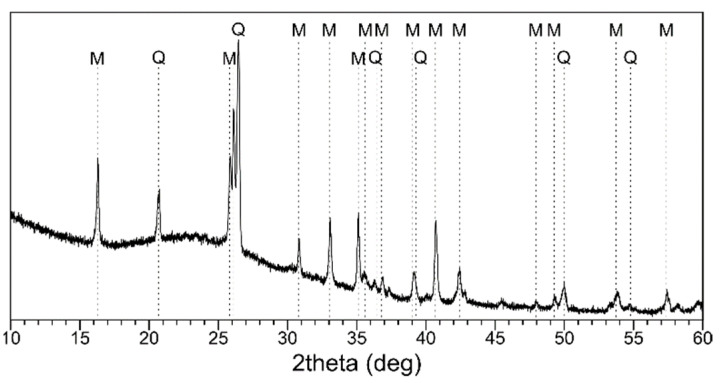
The XRD pattern of fly ash (M—mullite; Q—quartz).

The chemical composition of the FA is presented in Table 2. It was an aluminosilicate material (SiO_2_ and Al_2_O_3_ > 80 wt.%) of class F (according to ASTM C 618-05). Some heavy metals—cobalt, zirconium, chromium, zinc, nickel, copper, and lead—were present in trace amounts. An ‘amorphous halo’ can be seen in Figure 1, which suggests the high presence of glassy content. Moreover, the crystalline phases of quartz and mullite were identified.

### 3.2. Boroaluminosilicate Geopolymers

**Figure 2:**
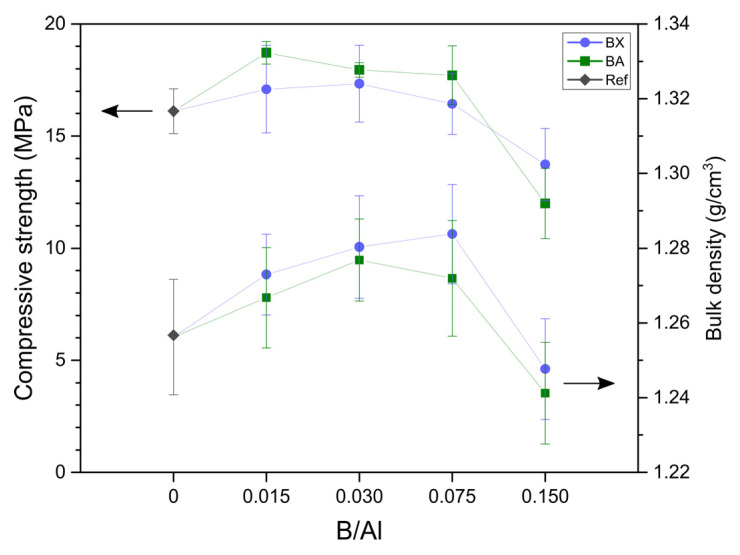
The compressive strength and bulk density of geopolymers with borax (BX) and boric acid (BA) as the source of boron (Ref—reference sample without boron).

**Figure 3:**
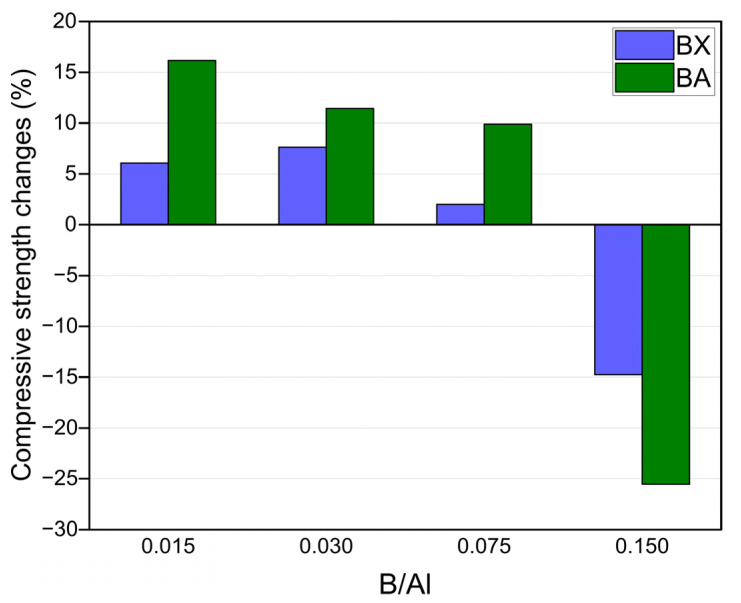
The relative changes in the compressive strength of geopolymers—the effect of the increasing boron content (in relation to geopolymer without boron).

Mechanical properties: The compressive strength and bulk density of the prepared geopolymers are presented in Figure 2. The compressive strength of the reference sample (without boron) was 16.1 MPa, and the addition of boron to the extent expressed in a B/Al molar ratio of 0.015–0.075 caused a slight increase in strength (16.4–17.3 MPa in the case of BX as the source of B and 17.7–18.7 MPa in the case of BA as the source of B). The values of the compressive strength of the samples with borax and boric acid were quite similar, so the effect of borax “water” on strength could be excluded. A higher content of boron (B/Al = 0.150) induced a decrease in the compressive strength to 13.7 MPa for BX and 12.0 MPa for BA (the relative changes of compressive strength due to the presence of B are shown in Figure 3). In [30], it was observed that replacing sodium silicate with borax led to a compressive strength drop from 45 to 30 MPa.

**Figure 4:**
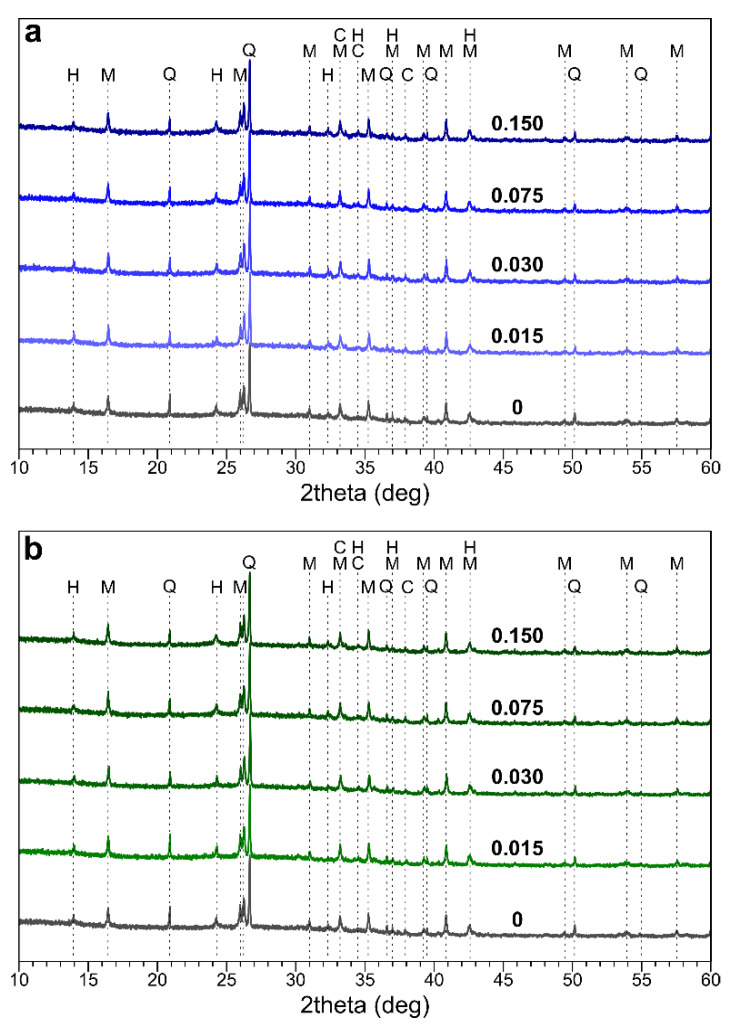
The XRD patterns of geopolymers with borax (**a**) and boric acid (**b**). 0—reference sample without boron (H—hydroxysodalite; M—mullite; Q—quartz; and C—sodium carbonates).

Phase composition: As can be seen in Figure 4, some crystalline phases, mainly quartz and mullite, were identified in the geopolymers; these were the unreacted components of the fly ash. The newly formed phases were sodium carbonates and zeolite-like feldspathoids, which could have been hydroxysodalite or hydroxycancrinite. Zeolite-like phases and zeolites are common phases that form during the geopolymerization process [34]. Sodium carbonates (e.g., trona, Na_3_H(CO_3_)_2_·2H_2_O, and thermonatrite, Na_2_CO_3_·H_2_O) formed due to the reaction between atmospheric CO_2_ and free sodium in the pore solutions [35,36]. There were no visible differences between the patterns of the samples with and without boron. This is consistent with [37], in which the presence of borax and boric acid did not lead to the appearance of new phases. It can be then concluded that boron was built into the structure of a geopolymeric N–A–S–H gel (N–A–S–H = Na_2_O·Al_2_O_3_·SiO_2_·H_2_O), thus forming N–B–A–S–H gel.

**Figure 5:**
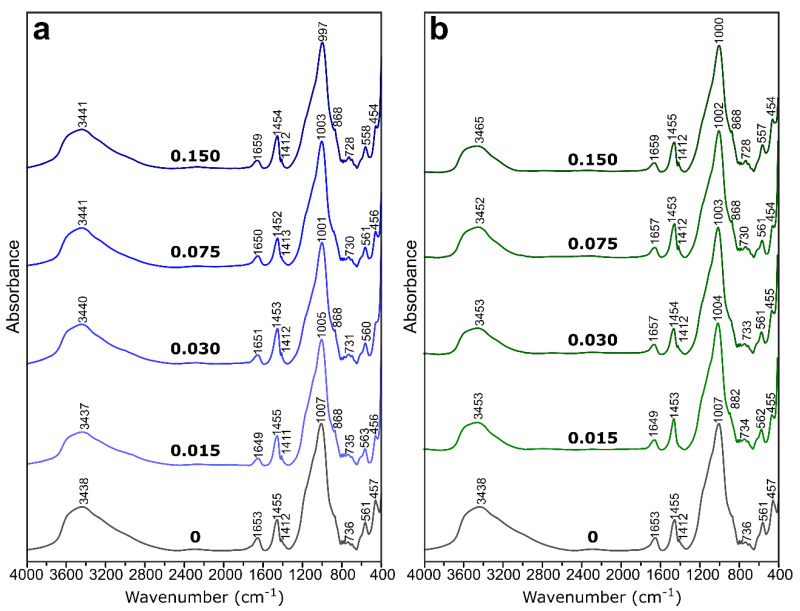
The FT-IR spectra of the geopolymers with borax (**a**) and boric acid (**b**). 0—reference sample without boron.

Structural studies: Figure 5 presents the infrared spectra of geopolymers with and without boron. In all of them, some groups of bands could be distinguished. The bands in the region of 3500–3400 cm^−1^ (stretching vibrations of O–H) and at around 1650 cm^−1^ (bending vibrations of H–O–H) were related to OH–groups and structural water molecules that were present in geopolymeric gel [38]. The bands at about 1450 and 860 cm^−1^ appeared due to the carbonation process, namely the stretching vibrations of C–O bond in the CO_3_ groups. The main band at 1000 cm^−1^ could be assigned to the vibrations of Si–O–Si and Si–O–Al bonds. There were no distinct changes in the spectra caused by the addition of boron compounds.

Generally, boron may occur in the structure of oxide glasses in both triangular and tetrahedral coordination. In the presence of alkali, it is more likely to change from three- to four-fold coordination without non-bridging oxygen formation [39]. Additionally, the introduction of aluminum reduces the amount of [BO_3_] groups in the glass [40]. Such a situation, namely the presence of alkalis and aluminum, existed in these geopolymeric systems and suggested that boron was present in their structure in a tetrahedral coordination. This was confirmed by the absence of a band at 1350 cm^−1^, which could have been related to the vibrations of boron in three-fold coordination.

Williams and van Riesen [27] stated that boron in borax, which is both three- and four-fold coordinated, is likely to reorganize during the reorientation stage of the reaction, and it results in a four-fold coordinated boron in a geopolymer. They had similar observations that the trigonal boron dissolved and rearranged into tetrahedral boron, which was introduced in the geopolymeric cross-linked network that was made by Taveri et al. [31]. The tetrahedral boron in the mixture could directly participate in the geopolymerization, resulting in enhanced polycondensation [37]. It should be noted that tetrahedra such as [AlO_4_] and [BO_4_] introduce additional negative charges to a structure that should play an important role in the heavy metal immobilization properties of boroaluminosilicate geopolymers.

The only response of the spectra to the increasing content of boron was a slight shifting of the main band towards lower wavenumbers from 1007 to 997 cm^−1^ in the case of BX and from 1007 to 1000 cm^−1^ in the case of BA. This may indicate that boron in the tetrahedral coordination was incorporated into the aluminosilicate network of geopolymers. In glass, B–O vibrations in [BO_4_] units were assigned to the band at 930 cm^−1^ [41], which should explain the shifting of geopolymeric band of 1007 cm^−1^ to lower wavenumbers in the presence of boron.

**Figure 6:**
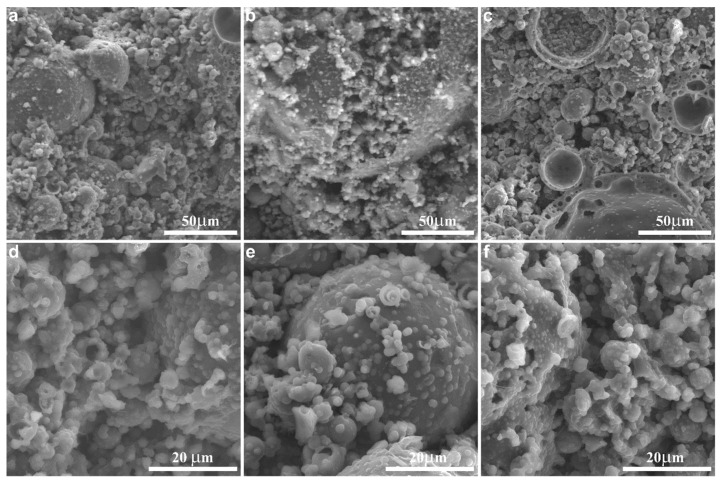
SEM images of the geopolymers reference sample (**a**,**d**), with BX (**b**,**c**) and BA (**e**,**f**). B/Al = 0 (**a**,**d**), 0.030 (**b**,**e**), and 0.150 (**c**,**f**).

Microstructure: SEM images of the geopolymers with and without boron are presented in Figure 6. The amorphous geopolymeric gel was visible in all samples. The spherical shapes in Figure 6a,e are fly ash particles that dissolved to a greater or lesser extent and that were covered with the condensed gel. Some concave voids of halved cenospheres (hollow particles of fly ash), partially filled with the geopolymerization products, are visible in Figure 6c. No crystalline products, as the result of boron addition, were observed. However, the microstructure of the samples with the highest boron content (Figure 6c,f) seemed less compact and more porous. Since the bulk density did not change much with the addition of boron compounds (remaining in the range of 1.24–1.28 g/cm^3^), the additional atoms in the structure must have increased the density. As such, the effect of structure relaxation caused by boron was evident.

### 3.3. Geopolymers with Heavy Metals

**Figure 7:**
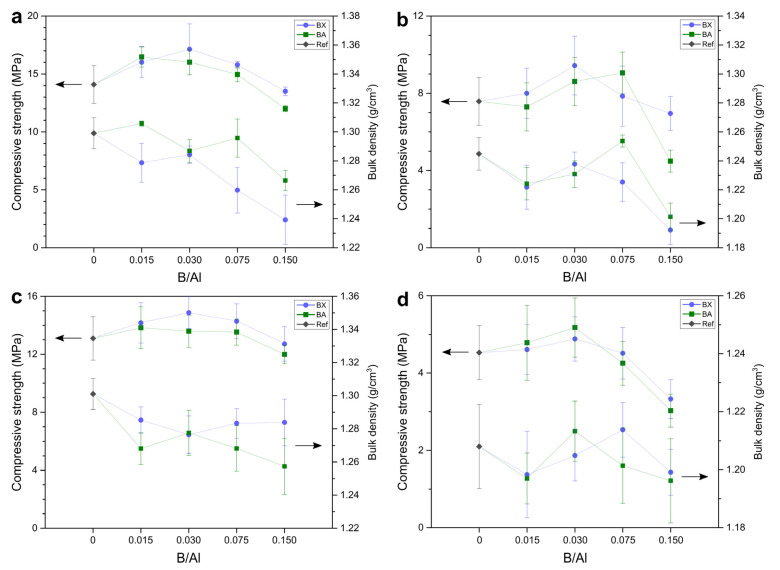
The compressive strength and bulk density of the geopolymers with borax (BX) and boric acid (BA) as the source of boron and (**a**) 2 wt.% of Pb^2+^, (**b**) 2 wt.% Ni^2+^, (**c**) 4 wt.% of Pb^2+^, and (**d**) 4 wt.% Ni^2+^ (Ref—reference sample without boron).

**Figure 8:**
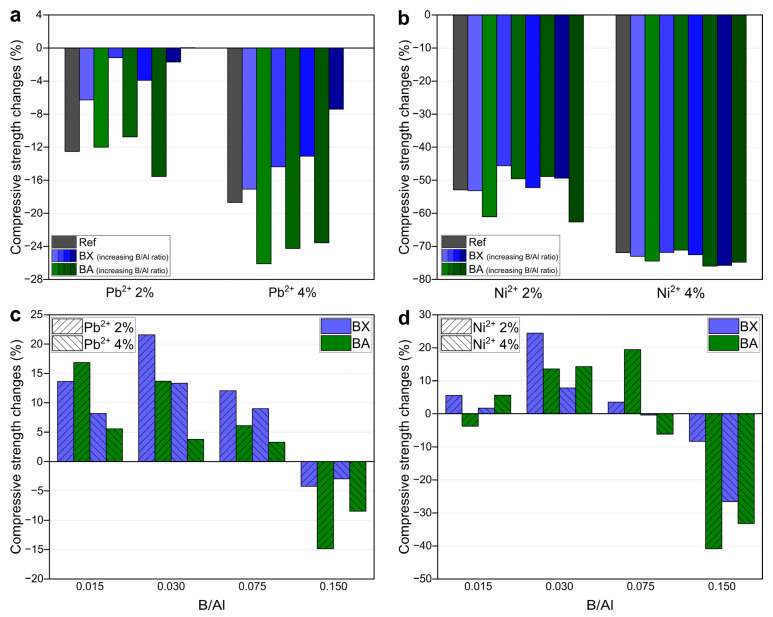
The relative changes in the compressive strength of geopolymers immobilizing heavy metals. The effect of the presence of (**a**) Pb^2+^ and (**b**) Ni^2+^ (in relation to the respective geopolymers without heavy metals). The effect of the boron presence (in relation to the geopolymer without boron) in geopolymers immobilizing (**c**) Pb^2+^ and (**d**) Ni^2+^.

Mechanical properties: Figure 7 presents the compressive strength and bulk density of the samples containing heavy metals, namely nitrate salts of lead and nickel. Their presence caused a decrease in the compressive strength of the geopolymers, both with and without boron. The reference samples without boron achieved about 14 MPa (2 wt.% of Pb^2+^), 12 MPa (4 wt.% of Pb^2+^), 8 MPa (2 wt.% of Ni^2+^), and 5 MPa (4 wt.% of Ni^2+^), while for sample without heavy metals achieved 16 MPa (Figure 2). As such, the relative strength loss was 12% and 18% caused by lead presence, and there was a much greater loss caused by nickel presence: 52% and 70% (Figure 8a,b). Lee et al. [42] observed a drop in compressive strength from 24 to 17 and 13 MPa, respectively for geopolymers with 0, 0.5, and 1 wt.% of Pb. This negative impact on compressive strength could have been related to not only heavy metal cations but also the nitrates that were introduced together with Pb^2+^ and Ni^2+^. Komnitsas et al. [43] stated that both nitrate and heavy metal ions influence compressive strength. Nitrate ions, even in low quantities, may prevent the hardening of a geopolymeric gel and, therefore, the development of compressive strength. The presence of a large quantity of nitrate is known to suppress silica solubility [44]. However, Nikolić et al. [45] showed that the addition of NaNO_3_ (2.4 wt.% of NO_3_^–^) had no effect on the compressive strength of geopolymers, while PbNO_3_ (the same amount of NO_3_^–^) caused an almost 25% decrease in compressive strength.

The loss of strength of the boroaluminosilicate geopolymers induced by Pb was significantly limited when B was introduced with borax (1–6% for 2 wt.% of Pb^2+^ and 7–17% for 4 wt.% of Pb^2+^); in the case of boric acid, the strength loss was even higher. However, in the case of Ni, there was no enhancement in the strength loss of the boroaluminosilicate geopolymers in comparison to that of the reference sample.

In Figure 8c,d, one can see a similar effect of the boron presence, like in the case of the samples without heavy metals (Figure 3) that had a slight increase in the compressive strength in the range of the B/Al ratio of 0.015–0.075 (but 0.030 for 4 wt.% of Ni^2+^). It can be stated that a certain amount of boron in the matrix of geopolymers has a positive impact on their compressive strength.

**Figure 9:**
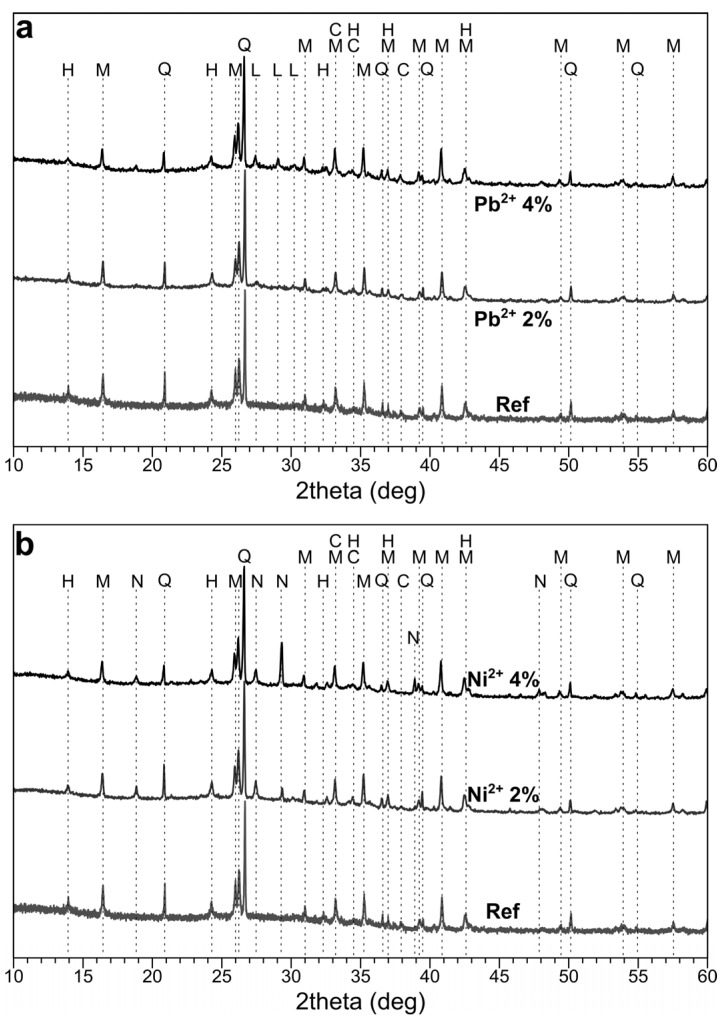
The XRD patterns of the geopolymers with heavy metals and without boron: (**a**) Pb^2+^ and (**b**) Ni^2+^ (H—hydroxysodalite; M—mullite; Q—quartz; C—sodium carbonate; L—lead oxide; N—nickel compounds; S—nitratine).

Phase composition: Several new peaks appeared in the diffraction patterns (Figure 9) as the result of heavy metal salt addition to the geopolymers. In the case of the samples with lead, the most matching phase was lead oxide, probably in the form of PbO·*x*H_2_O, which is likely to form in a highly alkaline environment. The addition of nickel nitrate caused the formation of sodium nitrate (nitratine) as the product of the reaction of NO_3_^–^ anions with Na^+^ cations from the alkaline activator. Minor amounts of some nickel compounds were also detected, most probably in the form of nickel hydroxide or silicate.

The formation of hydroxysodalite in the matrix of geopolymers may have had a positive impact on the immobilization of heavy metals. Ni^2+^ cations were immobilized in the center of a six-member ring of sodalite, while Pb^2+^ was six-fold coordinated by the oxygen atoms of the six-membered ring of the sodalite cage and three molecules of water [46].

**Figure 10:**
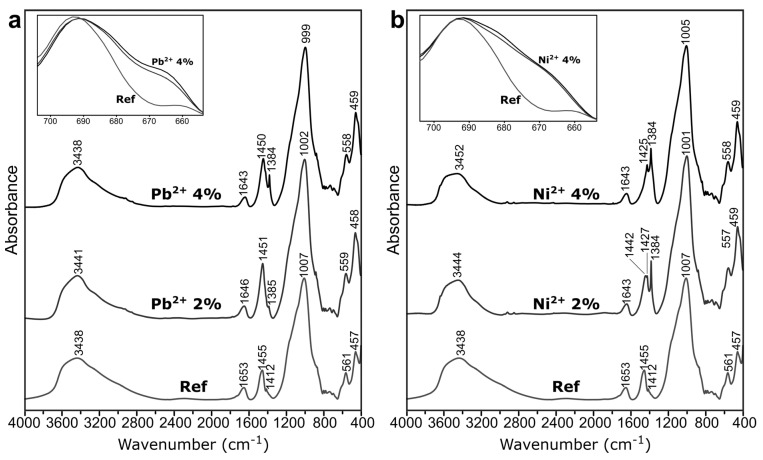
The FT-IR spectra of the geopolymers with heavy metals: (**a**) Pb^2+^ and (**b**) Ni^2+^.

Structural studies: The differences between the spectra of geopolymers with and without heavy metals (Figure 10) could be observed in three regions: about 1400, 1000, and 700 cm^−1^. The bands at 1384 cm^−1^ were related to nitrate ions [43,47], and their intensities increased with the higher dosage of heavy metal salts. The main band at about 1000 cm^−1^ slightly shifted to the lower wavenumbers in the presence of heavy metals. This might have been related to the formation of connections between geopolymeric frameworks (cross-linking) by heavy metal cations [48,49], but it is more likely that it was related to the increase in the non-bridging oxygen because the introduced heavy metal cations bound with non-bridging oxygen [50]. The last changes were observed in the range of 710–650 cm^−1^ (small images in Figure 10) related to pseudolattice vibrations (of overtetrahedral fragments of the structure). The change in the band intensity in this range could have been related to the ion-exchange of Na^+^ cations for heavy metal cations, since the ion-exchange induced an alteration in the ring’s surrounding, changes in ionic radii and charges of the cations, and the deformations of rings [51]. A similarity of the geopolymer network and the zeolite framework in terms of the occurrence of ring structures was indicated by Bortnovsky et al. [52], proving that a geopolymeric network is built of deformed 6-, 8-, and 10-membered rings.

**Figure 11:**
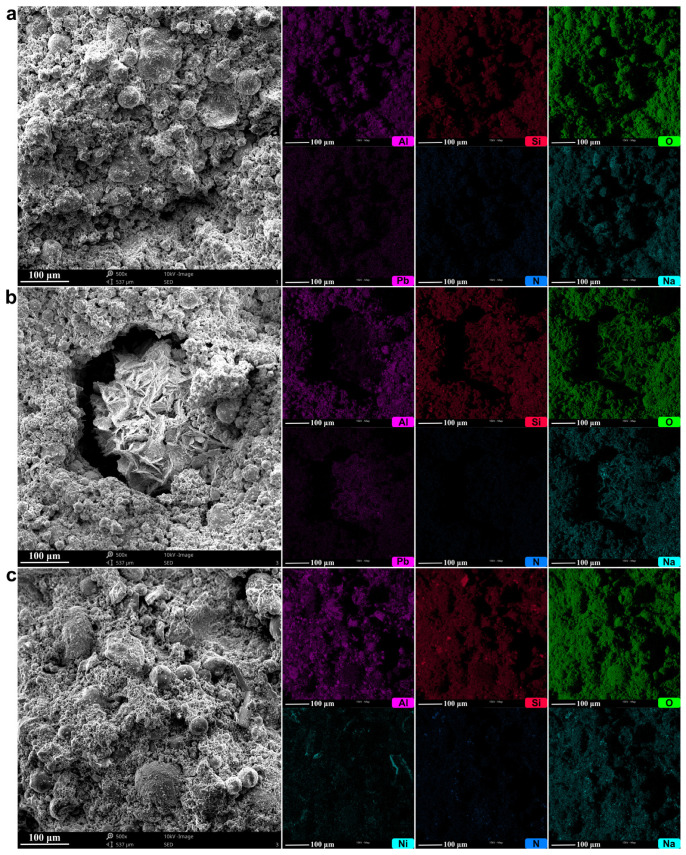
SEM images and EDS maps of the geopolymers with heavy metals: (**a**) the general image of the sample with Pb, (**b**) the inclusion in the sample with Pb, and (**c**) the general image of the sample with Ni.

Microstructure: SEM images and EDS maps of the samples with heavy metals are presented in Figure 11. There were no significant changes in the general microstructure caused by the presence of Pb (Figure 11a), though some sporadically occurring inclusions could be observed (Figure 11b). The elemental mapping of the general structure of the sample with lead showed that Si, Al, O, Na, and Pb were distributed regularly throughout the sample, which indicates the bonding of lead cations to the geopolymeric network (both ion-exchange and covalent bonding mechanism could be involved here). The crystalline, macroscopic inclusion shown in Figure 11b was composed of Pb, clearly to a greater extent than the rest of the sample, proving the presence of precipitation mechanism of immobilization and probably being PbO·*x*H_2_O. In the case of the sample with Ni, such macroscopic inclusions were not observed (Figure 11c), and only some microscopic regions with the higher nickel concentrations were visible.

**Figure 12:**
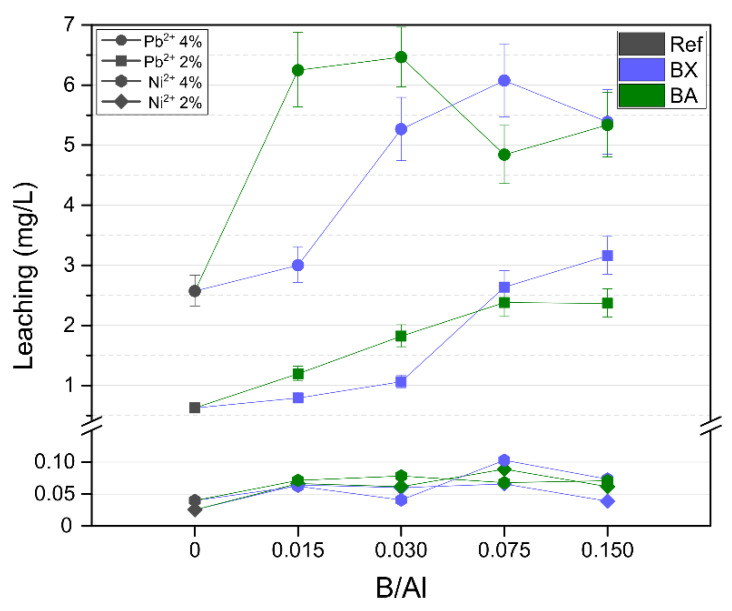
Heavy metal leaching from the geopolymers.

Immobilization properties: The values of heavy metals leaching from the geopolymeric samples are presented in Figure 12. In the case of the samples with 2 and 4 wt.% of Ni^2+^, the leaching was very low, namely < 0.05 mg/L for the reference sample (without boron). The leaching values were much higher for the reference samples with −0.6 mg/L (Pb^2+^ 2 wt.%) and 2.6 mg/L (Pb^2+^ 4 wt.%) of lead. The higher leachability of Pb than of Ni can be attributed to the occurrence of macroscopic crystalline inclusions of PbO·*x*H_2_O with a significant solubility in water. Another reason for the higher immobilization efficiency of Ni than Pb may be related to their exchange energies, which are higher for Pb^2+^ than Ni^2+^, and they are inversely related to solvation radii of heavy metals ions: Ni^2+^ (4.04 Å) > Pb^2+^ (4.01 Å)—the larger the solvation radius is, the more favorable the immobilization is [46]. Nevertheless, the immobilization rates (Table 3) were excellent. For the reference sample, they were >99.9% for Pb^2+^ and >99.99% for Ni^2+^. It should be noted that the heavy metal concentrations in the geopolymers were at extremely high levels, namely 20,000 and 40,000 mg/kg, but despite this, they were almost completely immobilized by the geopolymers. The typical contents in actual hazardous wastes, respectively, for Pb^2+^ and Ni^2+^ are: 398 and 90 mg/kg in municipal solid waste incineration fly ash [53], 1129 and 1420 mg/kg in municipal solid waste incineration bottom ash [54], 8966 and 50 mg/kg in waste glass from the spent fluorescent lamps [55], and 7060 and 93 mg/kg in sludge from wastewater treatment [56].

The presence of boron in the structure led to an increase in heavy metal leaching. This was probably because the addition of a boron compound to the geopolymers led to the creation of additional immobilization sites due to the formation of [BO_4_] tetrahedra, which provided more negative charges to the geopolymeric network and attracted heavy metal cations. However, the addition of boron caused an increase in geopolymer permeability, and this effect prevailed over the creation of additional immobilization sites, which resulted in an increased leachability. It should be noted that despite this, the immobilization rates of geopolymers with boron were still excellent: over 99.99% in the case of nickel and 99.84–99.96% in the case of lead. The presence of boron in real wastes subjected to alkali-activation can cause the increased leaching of some heavy metals, but its presence could also limit the compressive strength drop induced by heavy metal presence.

## 4. Conclusions

The addition of boron compounds to geopolymers slightly enhanced the compressive strength when the B/Al molar ratio was not higher than 0.075. A ratio of 0.150 caused a decrease in geopolymer strength. It was found that boron was incorporated into the geopolymeric structure and formed N-B-A-S-H gel. The boron presence did not induce the formation of any new crystalline phases. The immobilization properties of geopolymers were studied by introducing lead and nickel nitrates. A compressive strength drop was observed, and it was especially significant in the case of nickel addition. However, the boron presence in the matrix allowed us to limit the compressive strength decrease. Its presence had also a negative impact, because the heavy metal leaching increased with the increasing boron content. Nevertheless, the immobilization rates were excellent, especially when considering that heavy metals were added in extremal amounts of 20,000 and 40,000 mg/kg of fly ash. The immobilization rates in the case of the samples with lead were always higher than 99.8%, while in the case of the samples with nickel, the immobilization rates were higher than 99.99%.

## Figures and Tables

**Table 1:** Geopolymer composition. AA: alkali activator (NaOH) solution; FA: fly ash; BX: borax; BA: boric acid.

B/Al (mol/mol)	AA (mol/dm^3^)	AA/FA (g/g)	BX (wt.%)	BA (wt.%)
0	10	0.4	0	0
0.015			1	0.625
0.030			2	1.250
0.075			5	3.125
0.150			10	6.250

**Table 2:** The chemical composition of fly ash (FA).

Oxide Composition (wt.%):									
P_2_O_5_	SiO_2_	TiO_2_	Al_2_O_3_	Fe_2_O_3_	MgO	CaO	Na_2_O	K_2_O	LOI ^1^
1.1	51.4	1.2	33.8	4.5	1.6	1.6	1.1	2.3	4.7
**Trace Composition (mg/kg):**									
Co	Sr	Ba	Zr	Cr	Zn	Ni	Cu	Pb	As
2038	774	510	367	229	134	111	103	97	65

^1^ LOI = loss on ignition at 1000 °C.

**Table 3:** Heavy metal immobilization in the geopolymers.

Sample	B/Al	Immobilization Rate (%)			
		Pb^2+^ 2%	Pb^2+^ 4%	Ni^2+^ 2%	Ni^2+^ 4%
**Ref**	0	99.97	99.94	>99.99	>99.99
**BX**	0.015	99.96	99.93	>99.99	>99.99
	0.030	99.95	99.87	>99.99	>99.99
	0.075	99.87	99.85	>99.99	>99.99
	0.150	99.84	99.87	>99.99	>99.99
**BA**	0.015	99.94	99.84	>99.99	>99.99
	0.030	99.91	99.84	>99.99	>99.99
	0.075	99.88	99.88	>99.99	>99.99
	0.150	99.88	99.87	>99.99	>99.99

## Data Availability

The data presented in this study are available on request from the corresponding author.

## References

[1] Provis J.L., Duxson P., Van Deventer J.S.J. (2010). The role of particle technology in developing sustainable construction materials. Adv. Powder Technol..

[2] Duxson P., Provis J.L., Lukey G.C., van Deventer J.S.J. (2007). The role of inorganic polymer technology in the development of “green concrete”. Cem. Concr. Res..

[3] Zhang P., Zheng Y., Wang K., Zhang J. (2018). A review on properties of fresh and hardened geopolymer mortar. Compos. Part B Eng..

[4] Król M., Rożek P., Mozgawa W. (2017). Synthesis of the Sodalite by Geopolymerization Process Using Coal Fly Ash. Pol. J. Environ. Stud..

[5] Faisal M., Muhammad K., Gul S. (2016). Synthesis and characterization of geopolymer from bagasse bottom ash, waste of sugar industries and naturally available China clay. J. Clean. Prod..

[6] Hu W., Nie Q., Huang B., Shu X., He Q. (2018). Mechanical and microstructural characterization of geopolymers derived from red mud and fly ashes. J. Clean. Prod..

[7] Nie Q., Hu W., Huang B., Shu X., He Q. (2019). Synergistic utilization of red mud for flue-gas desulfurization and fly ash-based geopolymer preparation. J. Hazard. Mater..

[8] Sun Z., Vollpracht A. (2018). Isothermal calorimetry and in-situ XRD study of the NaOH activated fly ash, metakaolin and slag. Cem. Concr. Res..

[9] De Rossi A., Simão L., Ribeiro M.J., Novais R.M., Labrincha J.A., Hotza D., Moreira R.F.P.M. (2018). In-situ synthesis of zeolites by geopolymerization of biomass fly ash and metakaolin. Mater. Lett..

[10] Ojovan M.I., Lee W.E. (2011). Glassy wasteforms for nuclear waste immobilization. Metall. Mater. Trans. A Phys. Metall. Mater. Sci..

[11] Gougar M.L.D., Scheetz B.E., Roy D.M. (1996). Ettringite and C-S-H portland cement phases for waste ion immobilization: A review. Waste Manag..

[12] Lancellotti I., Ponzoni C., Barbieri L., Leonelli C. (2013). Alkali activation processes for incinerator residues management. Waste Manag..

[13] Liguori B., Cassese A., Colella C. (2006). Safe immobilization of Cr(III) in heat-treated zeolite tuff compacts. J. Hazard. Mater..

[14] Ji Z., Pei Y. (2020). Immobilization efficiency and mechanism of metal cations (Cd^2+^, Pb^2+^ and Zn^2+^) and anions (AsO_4_^3−^ and Cr_2_O_7_^2−^) in wastes-based geopolymer. J. Hazard. Mater..

[15] Nikolić V., Komljenović M., Džunuzović N., Ivanović T., Miladinović Z. (2017). Immobilization of hexavalent chromium by fly ash-based geopolymers. Compos. Part B Eng..

[16] Rożek P., Król M., Knapik A., Mozgawa W. (2017). Disposal of bottom ash from the incineration of hazardous waste in two different mineral matrixes. Environ. Prog. Sustain. Energy.

[17] El-eswed B.I. (2020). Chemical evaluation of immobilization of wastes containing Pb, Cd, Cu and Zn in alkali-activated materials: A critical review. J. Environ. Chem. Eng..

[18] Khater H.M., Ghareib M. (2020). Optimization of geopolymer mortar incorporating heavy metals in producing dense hybrid composites. J. Build. Eng..

[19] Zhao S., Xia M., Yu L., Huang X., Jiao B., Li D. (2021). Optimization for the preparation of composite geopolymer using response surface methodology and its application in lead-zinc tailings solidification. Constr. Build. Mater..

[20] Celik A., Yilmaz K., Canpolat O., Al-mashhadani M.M., Aygörmez Y., Uysal M. (2018). High-temperature behavior and mechanical characteristics of boron waste additive metakaolin based geopolymer composites reinforced with synthetic fibers. Constr. Build. Mater..

[21] Palomo A., López de la Fuente J.I. (2003). Alkali-activated cementitous materials: Alternative matrices for the immobilisation of hazardous wastes—Part I. Stabilisation of boron. Cem. Concr. Res..

[22] Nicholson C.L., Murray B.J., Fletcher R.A., Brew D.R.M., MacKenzie K.J.D., Schmücker M. (2005). Novel geopolymer materials containing borate structural units. World Congr. Geopolymer.

[23] Nazari A., Maghsoudpour A., Sanjayan J.G. (2014). Characteristics of boroaluminosilicate geopolymers. Constr. Build. Mater..

[24] Bagheri A., Nazari A., Sanjayan J.G., Rajeev P. (2017). Alkali activated materials vs geopolymers: Role of boron as an eco-friendly replacement. Constr. Build. Mater..

[25] Bagheri A., Nazari A., Hajimohammadi A., Sanjayan J.G., Rajeev P., Nikzad M., Ngo T., Mendis P. (2018). Microstructural study of environmentally friendly boroaluminosilicate geopolymers. J. Clean. Prod..

[26] Dupuy C., Gharzouni A., Sobrados I., Texier-Mandoki N., Bourbon X., Rossignol S. (2019). 29Si, 27Al, 31P and 11B magic angle spinning nuclear magnetic resonance study of the structural evolutions induced by the use of phosphor- and boron–based additives in geopolymer mixtures. J. Non-Cryst. Solids.

[27] Williams R.P., van Riessen A. (2011). Development of alkali activated borosilicate inorganic polymers (AABSIP). J. Eur. Ceram. Soc..

[28] Khezrloo A., Aghaie E., Tayebi M. (2018). Split tensile strength of slag-based boroaluminosilicate geopolymer. J. Aust. Ceram. Soc..

[29] Bagheri A., Nazari A., Sanjayan J.G. (2018). Fibre-reinforced boroaluminosilicate geopolymers: A comparative study. Ceram. Int..

[30] Bagheri A., Nazari A., Sanjayan J.G., Rajeev P., Duan W. (2017). Fly ash-based boroaluminosilicate geopolymers: Experimental and molecular simulations. Ceram. Int..

[31] Taveri G., Tousek J., Bernardo E., Toniolo N., Boccaccini A.R., Dlouhy I. (2017). Proving the role of boron in the structure of fly-ash/borosilicate glass based geopolymers. Mater. Lett..

[32] Bullerjahn F., Zajac M., Skocek J., Ben Haha M. (2019). The role of boron during the early hydration of belite ye’elimite ferrite cements. Constr. Build. Mater..

[33] Farid O.M., Abdel Rahman R.O. (2017). Preliminary assessment of modified borosilicate glasses for chromium and ruthenium immobilization. Mater. Chem. Phys..

[34] Rożek P., Król M., Mozgawa W. (2019). Geopolymer-zeolite composites: A review. J. Clean. Prod..

[35] Nath S.K., Maitra S., Mukherjee S., Kumar S. (2016). Microstructural and morphological evolution of fly ash based geopolymers. Constr. Build. Mater..

[36] Hajimohammadi A., van Deventer J.S.J. (2017). Solid Reactant-Based Geopolymers from Rice Hull Ash and Sodium Aluminate. Waste Biomass Valorization.

[37] Dupuy C., Havette J., Gharzouni A., Texier-Mandoki N., Bourbon X., Rossignol S. (2019). Metakaolin-based geopolymer: Formation of new phases influencing the setting time with the use of additives. Constr. Build. Mater..

[38] Król M., Minkiewicz J., Mozgawa W. (2016). IR spectroscopy studies of zeolites in geopolymeric materials derived from kaolinite. J. Mol. Struct..

[39] Stoch P., Stoch A. (2015). Structure and properties of Cs containing borosilicate glasses studied by molecular dynamics simulations. J. Non-Cryst. Solids.

[40] Stoch L., Środa M. (1999). Infrared spectroscopy in the investigation of oxide glasses structure. J. Mol. Struct..

[41] Adamczyk A., Handke M., Mozgawa W. (1999). FTIR studies of BPO4·2SiO2, BPO4·SiO2 and 2BPO4·SiO2 joints in amorphous and crystalline forms. J. Mol. Struct..

[42] Lee S., van Riessen A., Chon C.M., Kang N.H., Jou H.T., Kim Y.J. (2016). Impact of activator type on the immobilisation of lead in fly ash-based geopolymer. J. Hazard. Mater..

[43] Komnitsas K., Zaharaki D., Bartzas G. (2013). Effect of sulphate and nitrate anions on heavy metal immobilisation in ferronickel slag geopolymers. Appl. Clay Sci..

[44] Zhang J., Provis J.L., Feng D., van Deventer J.S.J. (2008). Geopolymers for immobilization of Cr6+, Cd2+, and Pb2+. J. Hazard. Mater..

[45] Nikolić V., Komljenović M., Džunuzović N., Miladinović Z. (2018). The influence of Pb addition on the properties of fly ash-based geopolymers. J. Hazard. Mater..

[46] Liu J., Luo W., Cao H., Weng L., Feng G., Fu X.-Z., Luo J.-L. (2020). Understanding the immobilization mechanisms of hazardous heavy metal ions in the cage of sodalite at molecular level: A DFT study. Microporous Mesoporous Mater..

[47] Ji Z., Pei Y. (2019). Geopolymers produced from drinking water treatment residue and bottom ash for the immobilization of heavy metals. Chemosphere.

[48] El-Eswed B.I., Aldagag O.M., Khalili F.I. (2017). Efficiency and mechanism of stabilization/solidification of Pb(II), Cd(II), Cu(II), Th(IV) and U(VI) in metakaolin based geopolymers. Appl. Clay Sci..

[49] Guo B., Pan D., Liu B., Volinsky A.A., Fincan M., Du J., Zhang S. (2017). Immobilization mechanism of Pb in fly ash-based geopolymer. Constr. Build. Mater..

[50] Hu S., Zhong L., Yang X., Bai H., Ren B., Zhao Y., Zhang W., Ju X., Wen H., Mao S. (2020). Synthesis of rare earth tailing-based geopolymer for efficiently immobilizing heavy metals. Constr. Build. Mater..

[51] Mozgawa W., Król M., Bajda T. (2009). Application of IR spectra in the studies of heavy metal cations immobilization on natural sorbents. J. Mol. Struct..

[52] Bortnovsky O., Dedecek J., Tvaružková Z., Sobalík Z., Subrt J. (2008). Metal Ions as Probes for Characterization of Geopolymer Materials. J. Am. Ceram. Soc..

[53] Luna Galiano Y., Fernández Pereira C., Vale J. (2011). Stabilization/solidification of a municipal solid waste incineration residue using fly ash-based geopolymers. J. Hazard. Mater..

[54] Rożek P., Król M., Mozgawa W. (2019). Solidification/stabilization of municipal solid waste incineration bottom ash via autoclave treatment: Structural and mechanical properties. Constr. Build. Mater..

[55] Bobirică C., Shim J.H., Park J.Y. (2018). Leaching behavior of fly ash-waste glass and fly ash-slag-waste glass-based geopolymers. Ceram. Int..

[56] Sun S., Lin J., Zhang P., Fang L., Ma R., Quan Z., Song X. (2018). Geopolymer synthetized from sludge residue pretreated by the wet alkalinizing method: Compressive strength and immobilization efficiency of heavy metal. Constr. Build. Mater..

